# Assessment of Phycocyanin Extraction from *Cyanidium caldarium* by Spark Discharges, Compared to Freeze-Thaw Cycles, Sonication, and Pulsed Electric Fields

**DOI:** 10.3390/microorganisms9071452

**Published:** 2021-07-06

**Authors:** Marie-Christine Sommer, Martina Balazinski, Raphael Rataj, Sebastian Wenske, Juergen F. Kolb, Katja Zocher

**Affiliations:** Leibniz Institute for Plasma Science and Technology, Felix-Hausdorff Straße 2, 17489 Greifswald, Germany; marie-christine.sommer@inp-greifswald.de (M.-C.S.); martina.balazinski@inp-greifswald.de (M.B.); raphael.rataj@inp-greifswald.de (R.R.); sebastian.wenske@inp-greifswald.de (S.W.); juergen.kolb@inp-greifswald.de (J.F.K.)

**Keywords:** phycocyanin, red algae, spark discharges, extraction, *Cyanidium caldarium*

## Abstract

Phycocyanin is a blue colored pigment, synthesized by several species of cyanobacteria and red algae. Besides the application as a food-colorant, the pigmented protein is of high interest as a pharmaceutically and nutritionally valuable compound. Since cyanobacteria-derived phycocyanin is thermolabile, red algae that are adapted to high temperatures are an interesting source for phycocyanin extraction. Still, the extraction of high quality phycocyanin from red algae is challenging due to the strong and rigid cell wall. Since standard techniques show low yields, alternative methods are needed. Recently, spark discharges have been shown to gently disintegrate microalgae and thereby enable the efficient extraction of susceptible proteins. In this study, the applicability of spark discharges for phycocyanin extraction from the red alga *Cyanidium caldarium* was investigated. The efficiency of 30 min spark discharges was compared with standard treatment protocols, such as three times repeated freeze-thaw cycles, sonication, and pulsed electric fields. Input energy for all physical methods were kept constant at 11,880 J to ensure comparability. The obtained extracts were evaluated by photometric and fluorescent spectroscopy. Highest extraction yields were achieved with sonication (53 mg/g dry weight (dw)) and disintegration by spark discharges (4 mg/g dw) while neither freeze-thawing nor pulsed electric field disintegration proved effective. The protein analysis via LC-MS of the former two extracts revealed a comparable composition of phycobiliproteins. Despite the lower total concentration of phycocyanin after application of spark discharges, the purity in the raw extract was higher in comparison to the extract attained by sonication.

## 1. Introduction

The bright blue colour of the pigment phycocyanin is of great interest as a natural colorant in food industry. Its good water solubility especially allows for the processing of extracted phycocyanin as a food colorant for ice cream, jelly or chewing gum [1]. Since commonly used colorants, such as Brilliant Blue FCF (E133) and Patent Blue V (E131) have been recently discussed as originators of allergic reactions [2,3], the interest in natural and sustainable alternatives is growing. Beyond the safe application of naturally derived phycocyanin as a food colorant, the pigment has been shown to have beneficial effects on health [4]. Phycocyanin acts as an anti-inflammatory agent and an antioxidant [5,6], and is therefore considered a potential drug for cancer treatment [7].

Most commonly, cyanobacteria such as *Arthrospira* sp. are used as phycocyanin source due to their widespread availability and cost-efficient cultivation [8]. The outstanding preference of *Cyanidium caldarium* to live in extreme habitats such as hot, acidic springs [9] qualify this red algae as an interesting alternative-producer of phycocyanin since colonization with contaminating microorganisms is unlikely due to the harsh culture conditions [10]. Furthermore, the pigments produced by extremophile red algae might possess different characteristics compared to the pigments exhibited by cyanobacteria. Red algae such as *Cyanidium caldarium* produce these accessory pigments in order to broaden the absorption spectrum of light used for photosynthesis. These pigments are characterised as phycobiliproteins [11] that can generally be divided into three groups: the reddish coloured phycoerythrin (PE), and the both blueish coloured C-phycocyanin (C-PC) and allophycocyanin (APC), all of them emitting a characteristic fluorescence after excitation with visible light [12,13]. C-phycocyanin is a holoprotein, which consists of two subunits [14]. The chromophore, phycocyanobilin, is attached to the apoprotein by a cysteinyl-linkage [15]. The fluorescence is quenched by denaturation of the protein or changes in the linkage between chromophore and apoprotein, which makes the stability of phycocyanin light and heat sensitive [16]. The small, spherical cells of *Cyanidium caldarium* possess a dense cell wall [17]. A successful extraction strategy needs to meet two challenges: the small size and the integrity of cell wall require several harsh disintegration processes, while the sensitiveness of phycocyanin limits the applied energy out of concern for the resulting high temperature. Different extraction methods have been described. For freeze-thaw-cycles, various protocols have been established [14,18,19]. Safaei et al. [18] examined different combinations of freeze-thaw-cycles for *Limnothrix* sp., indicating a three-times repeated freezing at −70 °C achieving the highest yields of phycocyanin. Major disadvantages of this method are the long treatment time, the high energy demand, and the number of freeze-thaw cycles strongly depending on the microorganism strain [19]. Other protocols are based on mechanical disruption by homogenisers [20,21]. Alternative methods include phycocyanin extraction after sonication of algae suspension [14] and the application of pulsed electric fields (PEF) for enhanced extraction of pigments from microalgae, e.g., chlorophyll, phycocyanin, and phycoerythrin [22,23,24]. The ultrasonic disintegration mainly derives from the formation of cavitation bubbles and the associated mechanical disruption of the cell walls. The most effective disruption frequency is dependent on the mechanical properties of the cell wall (such as thickness, composition and density) and specific for different algae species [25]. Generally, homogenisation or sonication often suffer from heat development, which might degrade thermolabile compounds. PEF, as a non-thermal technique for cell disintegration, charges the cell membrane, which eventually leads to pore formation and thus, increased permeability of cell membranes. This process of electroporation is utilised for the transfer of substances into a cell, as well as for increasing extraction yields of natural compounds from cells [26,27,28]. The increased extraction yields only occurred, if the treated samples were allowed to incubate after PEF exposure for at least two hours [24,29].

Spark discharge treatment of algae suspensions has been shown to be an effective, but gentle tool for the disintegration of microalgae and with regard to pigment and protein extraction [30,31,32]. In general, spark discharges generated in aqueous solution provide ultraviolet radiation and shockwaves besides a variety of chemical reactive species, e.g., hydrogen peroxide, hydrogen, oxygen, superoxide or hydroxyl radicals [33]. Importantly, the effect of successful cell wall disintegration is mainly based on the generation of shockwaves with a pressure of approx. 500 MPa, which usually exceeds the tensile strength of most if not all microalgae [32]. With the applied parameters, the bulk temperature did not exceed 27 °C, which are optimal extraction conditions for heat sensitive pigments.

The aim of this study was to determine the efficiency of spark discharges compared to standard treatment protocols, e.g., freeze-thaw cycles, pulsed electric fields, and sonication. The results were evaluated in respect of scaling-up potential. To keep the comparability of all applied techniques, the total energy input was kept constant, except for freeze-thaw cycles. To examine the extracted phycocyanin for protein modifications caused by the disintegration method, protein analysis via LC-MS was performed for selected extracts.

## 2. Materials and Methods

### 2.1. Cultivation and Harvesting

Algae suspension of *Cyanidium caldarium* was used for all experiments. The algae were maintained in a modified *Galdieria*-medium in 100 L scale at 34 °C under continuous light (100 µmol/m^2^s) and harvested 21 days after inoculation, exhibiting a dry biomass equivalent of approximately 4 g/L. After harvesting, the biomass was concentrated by centrifugation to a dry biomass equivalent of 100 g/kg. The pH of the suspension was adjusted with sulphuric acid to 2.1, and the conductivity was 2.3 mS/cm.

For each of the individual conditions, a volume of 75 mL algae suspension was subjected to disintegration. Experiments were performed in triplicate.

### 2.2. Disintegration by Freeze-Thaw-Cycles

Freeze-thaw cycles were performed in accordance to the procedure suggested by *Safaei* et al., with some modifications. First, the algae suspension was lyophilised (Christ Alpha 1–4 LSC, Martin Christ Gefriertrocknungsanlagen GmbH, Germany). Subsequently, the dry biomass was resuspended in distilled water to the original volume of 75 mL and freeze thaw cycles were repeated three times. Freezing was performed at −70 °C for 24 h, followed by thawing the suspension at 4 °C for another 24 h. Without accounting for losses in the cooling device, the estimated energy input for all three cycles was 111,900 J in total.

### 2.3. Disintegration by Sonication

The algae suspension was exposed to a sonicator (Q700, Qsonica) in a 100 mL beaker. The sonotrode was placed in the centre of the algae suspension, approximately 2 cm above the beaker ground. The amplitude was set to maximum, i.e., 120 μm elongation at a frequency of 20 kHz, resulting in a provided sonication power of 114 W. The algae suspension was treated for 1 min 44 s, which equals an energy dissipation of 11,880 J in the sonicator. Due to strong heating of the suspension, the treatment was conducted in two different ways. The total energy input was either applied at once (continuous sonication) or sonication was paused after each 30 s until the temperature of the suspension was <20 °C (intermittent sonication).

### 2.4. Disintegration by Pulsed Electric Fields (PEF)

Two plasma-polished stainless steel electrodes with a diameter of 32 mm were placed in parallel with a distance of 4 mm into a 3.22 µL treatment chamber as described by Eing et al. and Goettel et al. [34,35]. The treatment was carried out by a continuous-flow system with a flow rate of 100 mL/min. The algae suspension was treated for 30 min at a frequency of 15 Hz with similar 100 ns pulses as applied for the spark treatment, but only a mean pulse amplitude of 14 kV (≙35 kV/cm field strength). Because of the differences in electrode geometry, lower pulse amplitude, and greater gap distance, no discharges were encouraged in this configuration. Average pulse energy was determined with 0.44 J, which corresponds to 11,880 J for a complete treatment.

### 2.5. Disintegration by Spark Discharges

**Figure 1:**
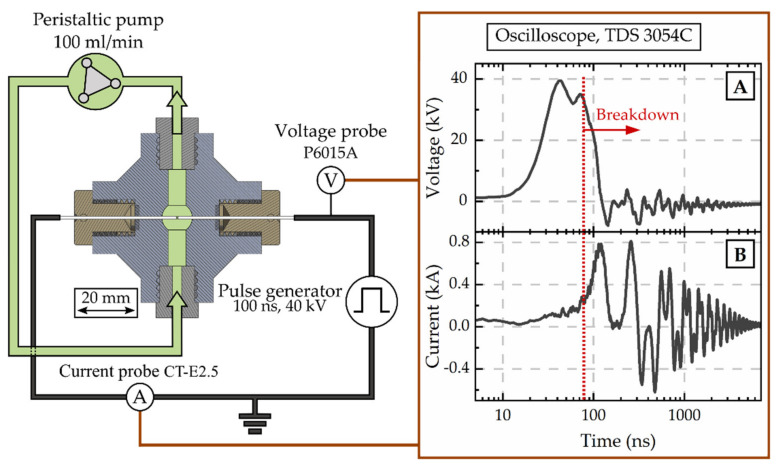
Schematic of the setup for the disintegration by spark discharges. The discharge chamber, algae pumping loop (green) as well as the electric circuit (black) with diagnostics, are displayed and typical voltage (**A**) and current (**B**) measurements are shown.

Spark discharges were ignited in the algal suspension between the tips of two tungsten rods with a diameter of 1 mm and a gap distance of 0.5 mm. As schematically visualised in Figure 1, the suspension was recirculated through a spherical treatment chamber of 520 μL with a flow rate set to 100 mL/min (peristaltic pump BT300-2J, Longer Precision Pump Co., Ltd., Hebei, China). Sparks were applied for 30 min with a frequency of 11 Hz. The sparks were ignited with the electrical breakdown of 100 ns high voltage pulses of 40 kV that were applied from a custom-build Blumlein pulse forming network. Typically, pulse energies of 0.6 J were measured resulting in total energy dissipation of 11,880 J. Voltage and current signals were measured with a passive high-voltage probe (P6015A, Tektronix Inc., Beaverton, OR, USA) as well as a Rogowski coil (Model 5046, Pearson Electronics, Palo Alto, CA, USA), respectively, and were recorded with an oscilloscope (TDS 3054C, Tektronix Inc., Beaverton, OR, USA). Typical signals are shown in Figure 1 and the breakdown is indicated.

### 2.6. Photometric Determination of Phycocyanin Content and Purity of Raw Extract

After the disintegration, the suspension was centrifuged at 3000× *g* for 10 min at room temperature. The supernatant was transferred to a new falcon and centrifugation was repeated. The remaining supernatant was taken as the raw extract and used for photometric evaluation (Infinite^®^ M200 PRO Tecan Platereader, Basel, Switzerland) of phycocyanin content and purity, as well as for protein precipitation for further characterization.

Calculations were performed as described by Abalde et al. and Bennett and Bogorad et al. [14,36]. A volume of 100 µL supernatant was analysed in a 96-well-plate, and the absorption was detected at 615 nm, 652 nm, and 280 nm, respectively. The absorption of pure water was subtracted from each sample. The amount of extracted phycocyanin (*PC*) was calculated according to Equation (1) [36]:

(1) $$PC=\frac{OD_{615}-0.474\cdot OD_{652}}{5.34}$$

Purity (*EP*) was estimated by the ratio of absorption at 615 nm and the amount of protein, given by the absorption at 280 nm, according to Equation (2) [14]:

(2) $$EP=\frac{OD_{615}}{OD_{280}}$$

### 2.7. Protein Precipitation and Desalination

An amount of 13 mg ammonium sulphate was added to the raw extract and mixed vigorously to then precipitate phycocyanin over night at 4 °C. The precipitated raw extract was stored in ammonium sulphate solution at 4 °C until further use. After centrifugation (3000× *g*, 10 min, room temperature), the supernatant was discarded and the pellet was dissolved in 1 mL water (LC-MS-grade). The desalination was performed with PD-10 desalting columns (GE Healthcare, Danderyd, Sweden), following the manufacturer’s instructions. The purified extract was analysed by fluorescence and absorption spectroscopy, and the protein fingerprint of phycocyanin was studied with LC-MS/MS-based protein analyses.

### 2.8. Evaluation of the Purified Extract by Fluorescence-Spectroscopy

Fluorescence spectra of 1 mL purified extract were obtained with a Fluorolog^®^ spectrometer (Horiba Jobin Yvon GmbH, Bensheim, Germany). Excitation wavelength was set to 620 nm and the emission was detected in a range of 600 nm to 750 nm with an increment of 0.5 nm. Spectra were recorded in triplicates.

### 2.9. Evaluation of Purified Extract by Absorption Spectroscopy

Absorption spectra were obtained with a spectrophotometer (Evolution 300, ThermoFischer Scientific, Madison, WI, USA). The absorption was recorded in a range of 250 nm to 700 nm. Spectra were recorded in triplicates.

### 2.10. NanoLC-MS/MS Data Acquisition and Protein Identification

For sample preparation, 25 µL trypsin with a concentration of 20 µg/mL (Promega, Madison, WI, USA) were added to an aliquot of 100 µL of the purified extract. Digestion was performed for 20 h at 37 °C. Nanoflow liquid chromatography mass spectrometry (nanoLC-MS/MS) was performed with an UltiMate 3000 RSLCnano system (Thermo Scientific, Madison, WI, USA) with an Acclaim Pepmap C18 column (150 mm × 75 µm, 2.0 µm particle size,) and the corresponding precolumn (20 mm × 100 µm, 5.0 µm particle size; both Thermo Scientific, Madison, WI, USA) for desalting and cleaning of the sample. The injection volume for each sample was 1 µL, corresponding to 0.02 µg protein. The gradient for sample separation (see Table 1) consisted of water with 0.1 % *v*/*v* acetic acid (A) and acetonitrile with 0.1 % *v*/*v* acetic acid (B), both LC-MS-grade (Merck KGaA, Darmstadt, Germany).

Mass spectrometry (MS) acquisition was carried out in a data dependent mode with a full scan range of *m*/*z* 300 to 1650 (resolution 70,000), and with the fragmentation of the top 15 most abundant precursor ions (QExactive Hybrid-Quadrupol-Orbitrap, Thermo Scientific, Madison, WI, USA). Precursor ions were fragmented by using a normalized higher-energy collisional dissociation (27.5 eV) and were detected at a mass resolution of 17,500 at *m*/*z* 200. The automatic gain control target for full MS was set to 6.37 × 10^6^ ions and for MS/MS to 2e^5^ ions with a maximum ion injection time of 100 ms for both modes. A dynamic exclusion was set to 60 s and ions with a charge state one, or above seven were excluded. To identify the peptides and potential post-translational modification, the MS raw data were analysed with the software Proteome Discoverer 2.4 (Thermo Scientific, Madison, WI, USA). The modifications in the amino acid chain of the peptides were scanned with the software Byonic Version 3.6.0 (Protein Metrics, Cupertino, CA, USA), as a plug-in tool for the Proteome Discoverer. Detected modifications were filtered with a Byonic score > 250 and Delta Mod score > 5.

Protein data for the phycobiliproteins (Allophycocyanin (APC), C-phycocyanin (C-PC), R-phycocyanin (R-PC), and phycoerythrin (PE)) were taken from Uniprot^®^ database [37]. The protein composition of the attained extracts was compared to reference phycobiliproteins of *Arthrospira platensis* (A. s.), *Cyanidium caldarium* (C. c.), *Galdieria sulphuraria* (G. s.), *Porphyridium purpureum* (P. p.), *Polysiphonia urceolata* (P. u.), and *Synechococcus* sp. (S. sp.). Abundances are given as averages of duplicates obtained by MS-protein analysis.

## 3. Results

Phycobiliprotein extractability of *Cyanidium caldarium* with standard extraction methods and spark discharges was determined by absorption and fluorescence spectroscopy with respect to phycocyanin, allophycocyanin and phycoerythrin content released to the supernatant. For freeze-thaw cycles, pulsed electric fields (PEF), and sonication with fixed temperature no increase in phycocyanin yield was detected, compared to untreated controls. The application of spark discharges resulted in a moderate increase of phycobiliprotein content, while sonicated samples showed a steep increase, when the total energy was applied at once. Protein analysis revealed that, independent of the disintegration method, the composition of phycobiliproteins is divers, including *C*-phycocyanin, allophycocyanin, R-phycocyanin, and phycoerythrin. Based on the rate of modifications in the amino acid chain of the peptides, the spark discharge showed to be the gentlest procedure with least modifications. Images of the raw extracts from freeze-thawing, sonication and spark discharge treatment can be found in the Supplemental Section (Figure S1).

### 3.1. Freeze-Thaw Cycles

**Figure 2:**
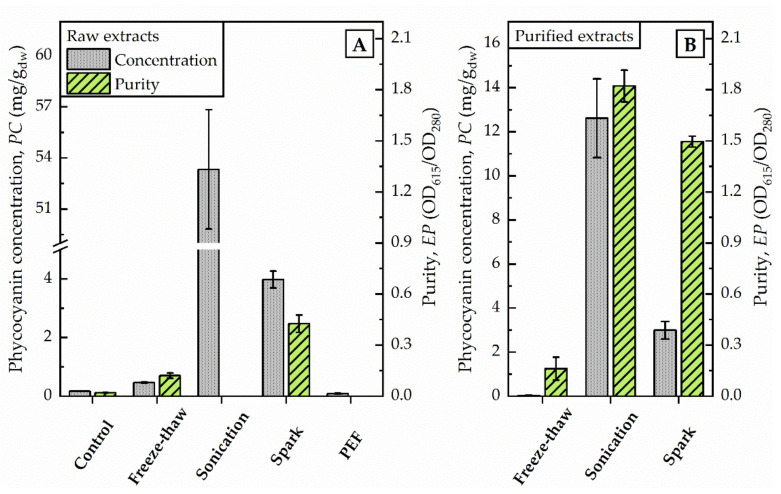
Photometric determination of raw extracts (**A**) and selected purified extracts (**B**) after different disintegration methods. Phycocyanin concentration (PC) and purity (EP) were calculated as given in Equations (1) and (2) regarding to Bennett and Bogorad, and Abalde et al., respectively. PC is given in mg/g dried weight (dw) of biomass (100 g/L).

By increasing the volume (from 1.5 mL to 75 mL) of algae suspension conducted to freeze-thaw-cycles at once, the supernatant after centrifugation remained colourless although an order of magnitude more energy, 111,900 J was dissipated. The phycocyanin content estimated by photometric determination did not differ from untreated control (0.2 mg/g dw) and was calculated with 0.5 mg/g dw (see Figure 2). The evaluation of the purified extract by fluorescence spectroscopy supported this finding. The characteristic fluorescence maximum after excitation at 620 nm was absent in the samples (data not shown). Still, the elevated absorption of freeze-thaw samples at 280 nm indicated that proteins were extracted from the algae.

### 3.2. Sonication

The disintegration by sonication was divided into two different treatment schemes. First, the total energy (11,880 J) of the sonication process was applied at once, leading to a rise in temperature of the algae suspension above 45 °C. In order to avoid possible damages caused by the high temperature, the sonication process was paused after each 30 s in a second treatment scheme, allowing the algae suspension to cool down intermittently to 20 °C. By this process, the maximum temperature did not exceed 27 °C, comparable to the temperatures obtained after disintegration by spark discharges.

#### 3.2.1. Continuous Sonication

**Figure 3:**
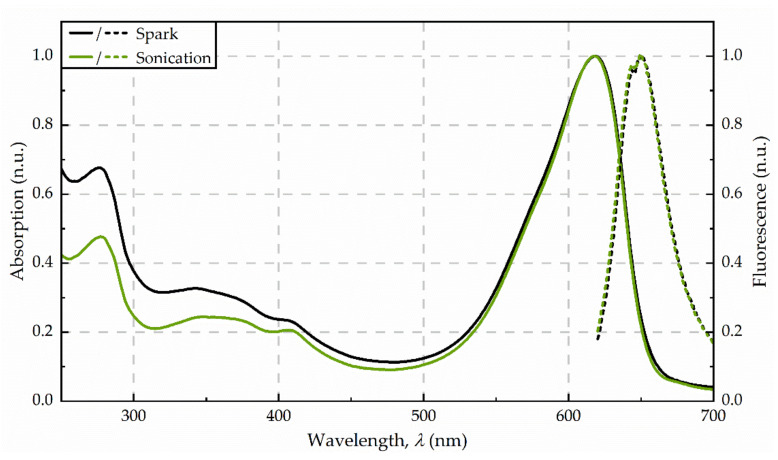
Absorbance (solid line) and fluorescence (dotted line) spectra from the purified extracts, obtained for disintegration by spark discharges and sonication, respectively. Spectra were averaged from three replicates and normalized to 620 nm (absorption) as well as 650 nm (fluorescence).

The supernatant after centrifugation was of a dark blue colour. The photometric determination of phycocyanin content indicated a concentration in the raw extract of 53 mg/g dw (see Figure 2). The maximum absorbance was detected at 618 nm. Precipitation with ammonium chloride revealed a green-blue pellet. The purified extract was of a bright blue colour. Analysis of fluorescence spectra shows a fluorescence maximum at 650 nm after excitation with 620 nm (see Figure 3).

#### 3.2.2. Intermittent Sonication

Limiting the rise of temperature by pausing of sonication process led to a marginal release of phycocyanin (data not shown). The supernatant after centrifugation was of a dark green colour without visible traces of blue. Since the high amount of impurities inhibited the purification, no further evaluation of phycocyanin characteristics were examined.

### 3.3. PEF Exposure

The application of pulsed electric fields with a total energy of 11,880 J did not foster the release of phycocyanin from algae cells. The phycocyanin concentration in the raw extract corresponded to values of untreated controls, as illustrated in Figure 2. The photometric determination of phycocyanin concentration was calculated with 0.09 mg/g dw and is in good agreement with the visual evaluation. The raw extract appeared colourless without traces of blue colour.

### 3.4. Spark Discharge Treatment

After the exposure of the algae cells to spark discharges in a continuous flow-through system, the raw extract was of an intense blue colour. The photometric determination of phycocyanin concentration showed a distinct deviation from untreated control. Compared to the phycocyanin concentration in the raw extract after sonication, the concentration was 13-times lower (≙ 4 mg/g dw) after disintegration by spark discharges. Detailed analysis of absorbance and fluorescence spectra of the purified extract revealed an absorbance maximum at 619 nm and a fluorescence maximum at 650.5 nm (see Figure 3). In the purified extract, the ratio of phycocyanin yield was four times lower, when comparing spark discharge (3 mg/g dw) to sonication (13 mg/g dw).

### 3.5. Protein Analysis

**Figure 4:**
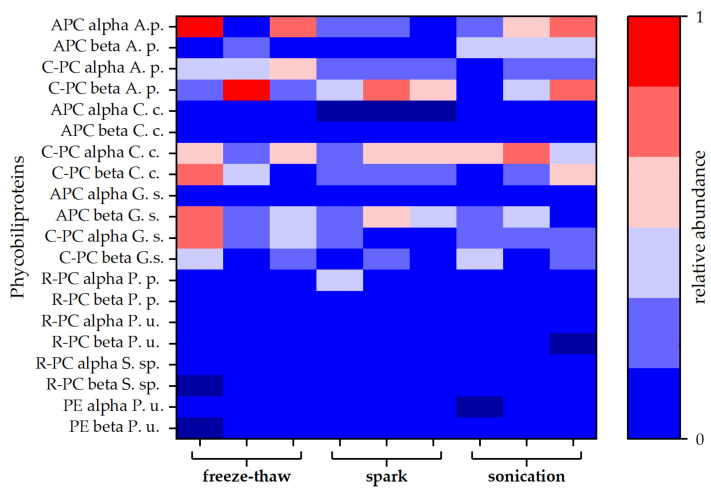
Relative abundances of different types of phycobiliproteins. Each disintegration method was performed in triplicate. Abundances are given as averages of duplicates obtained by MS-protein analysis. Allophycocyanin (APC), C-phycocyanin (C-PC), R-phycocyanin (R-PC), and phycoerythrin (PE) sequences of different species were obtained from the Uniprot^®^ database. Protein composition of the attained extracts was compared to phycobiliproteins of *Arthrospira platensis* (A. s.), *Cyanidium caldarium* (C. c.), *Galdieria sulphuraria* (G. s.), *Porphyridium purpureum* (P. p.), *Polysiphonia urceolata* (P. u.), and *Synechococcus* sp. (S. sp.).

For a more detailed study of the extracted proteins, a protein analysis via LC-MS was carried out for selected extracts. Both blue coloured extracts (spark discharge and sonication) were compared to the extract obtained by freeze-thaw cycles, which served as reference method. Independent of the disintegration method, different phycobiliproteins were found in all samples. Besides allophycocyanin (APC) and C-phycocyanin (C-PC) protein subunits alpha and beta, lower amounts of R-phycocyanin (R-PC) and phycoerythrin (PE) were identified. Interestingly, the amount of APC was lower compared to C-PC for all types of disintegration. Particularly, after disintegration by spark discharges, no APC alpha chain was found in the samples (see Figure 4).

**Figure 5:**
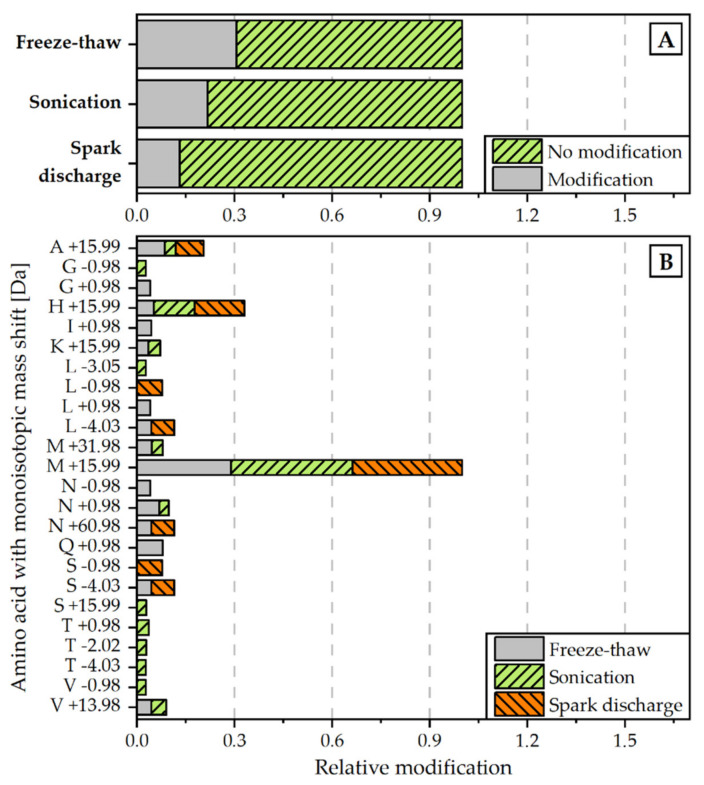
Relative amount of modifications detected for each disintegration method (normalized to all peptide spectrum matches) (**A**) and monoisotopic mass shifts [Da] for specific amino acids (normalized to overall amount of M +19.99 modification) (**B**). Modifications were scanned with the Byonic Version 3.6.0 from Proteome Discoverer^®^ 2.4. Detected modifications were filtered with a Byonic Score > 250 and Delta Mod Score > 5.

In a second approach, the proteins were examined with regard to non-enzymatic modifications in the amino acid chain of the peptides. The analysis revealed that the relative rate of modifications of target proteins is the highest for the extract, obtained by freeze-thaw cycles. The proteins extracted with the help of spark discharges underwent the least modifications (see Figure 5A). The most frequent detected modification for all disintegration variants was M +15.99, which is associated with the oxidation of methionine (see Figure 5B).

## 4. Discussion

*Cyanidium caldarium* as extremophile alga is an interesting alternative source for phycocyanin, also in comparison to other algae and cyanobacteria, e.g., *Arthrospira platensis*. Usually, phycocyanin is extracted by freeze-thaw cycles with various protocols. However, the method is associated with disadvantages, such as long treatment times, strong thermal effects on the extractives, and a high energy consumption. In this work, spark discharges were applied as an innovative tool and evaluated for their potential with respect to the extraction of phycocyanin from microalgae compared to freeze-thawing as reference and other physical methods (PEF and sonication). The total energy input for every physical method was kept the same as a basis for a process-relevant comparison. In addition, high biomass concentrations were used to mimic realistic industrial conditions. Where applicable, scaling recommendations are given.

Freeze-thaw cycles are often used to extract phycocyanin from cyanobacteria due to the in principle simple procedure [19]. In this work, a freeze-thaw protocol was adapted from Safaei et al. [18] who reported that freezing at −70 °C for 4 h and thawing at 4 °C for another 4 h, repeated in triplicate, resulted in the best results for phycocyanin extraction (11.25 mg/g dw) from the cyanobacterium *Limnothrix* sp. In this study, the application of freeze-thaw cycles to the red algae *Cyanidium caldarium* achieved only values of 0.5 mg/g dw (see also Figure 2). On the one hand, it is likely that *Cyanidium caldarium* comprises a much stronger cell wall than the aforementioned cyanobacteria and the temperature stress is not enough to rupture the cell walls effectively [17,38]. On the other hand, freeze-thawing was conducted with a cell density of 100 g/mL, which is a realistic condition for industrial downstreaming, but much higher than in other protocols described. It might be possible that higher cell densities are less affected by this method than rather low cell densities. However, either providing harsher temperature stress (e.g., lower freezing temperature) or reducing the cell densities, would limit the practical relevance further. Also, even for a single freeze-thaw cycle, an energy of 37,300 J is required, limiting the achievable efficiency compared to other investigated disintegration methods. Therefore, any change of these parameters was not pursued for the conducted comparison.

Sonication was the most effective extraction method in this study. An amount of 53 mg/g dw of phycocyanin in the raw extract was obtained from *Cyanidium caldarium*. However, these results are only valid when the total energy was applied in one step, resulting in a steep increase in temperature to above 45 °C. This finding is in good agreement with the conclusion of Hadiyanto et al. [39] who demonstrated a temperature optimum over 50 °C for ultrasound-based phycocyanin extraction from *Arthrospira platensis*. The temperature seems to have a major impact on the amount of phycocyanin extracted after disintegration by ultrasound. While the yield of high quality phycocyanin after the application of the whole energy of 11,880 J by sonication in one step was high, only small amounts of phycocyanin with poor quality were extracted for intermittent treatment together with cooling to keep the temperature below 28 °C. Li et al. [40] demonstrated that sonication of dried *Arthrospira platensis* under cooled conditions increased the yield of phycocyanin; nevertheless, chlorophyll impurities strongly decreased the purity pf phycocyanin and was thus not recommended by the authors. Fratelli et al. [41] note in their review that heat development is the major drawback of ultrasound application for phycocyanin extraction, despite the efficiency. Heat-related protein denaturation was responsible for activity loss of phycocyanin. In addition, energy consumption can be very high.

Pulsed electric fields were already successfully applied for the extraction of phycobiliproteins from cyanobacteria and red microalgae. Martinez et al. [22,23] achieved phycocyanin extraction yields between 6.5 to 159.9 mg/g dw of small concentration and low volume *A. platensis* culture (150 µL algae suspension with a concentration of 1 g/L) after 360 min treatment time, when 15 to 50 pulses of 3 µs were applied to the suspension. However, it was found that an incubation time, subsequent to the end of the treatment, of at least 150 min was required to detect phycocyanin in the sample solution. For phycoerythrin extraction from *Porphyridium cruentum*, the authors found an incubation time of at least 6 h was necessary to detect noteworthy amounts of phycoerythrin and achieved the highest yields after 24 h of incubation time. A possible explanation could be that the phycobiliproteins do not only have to cross the cell membrane, but also dissociate from the thylakoid membrane. In this study, no phycocyanin was detected after 120 min incubation time, which is comparable to the results of Martinez et al. Additionally, Akaberi et al. [42] found a decrease in phycocyanin release and increase in incubation time (from 1 h to 5 h) when the biomass density was raised from 3.6 g/kg dw to 9.7 g/kg dw. It was concluded that release kinetics at high biomass concentration are significantly slower compared to low applied biomasses. However, long incubation times between treatment and further downstreaming, as well as a dependency on utilised biomass concentration, decrease the economic applicability of phycocyanin extraction: especially as continuous sonication and spark discharges show significant extraction yields for comparable energy input. Thus, PEF may not be a recommended technique for the extraction of this pigment on an industrial scale.

Spark discharges have been found to be effective against stiff cell walls due to the development of strong shockwaves [32]. Accordingly, it could be demonstrated that proteins and pigments, e.g., chlorophylls and carotenoids from *Chlorella vulgaris* are well extractable with this technique. Moreover, these often heat-sensitive compounds were not affected by the sparks [30,31]. Also, Zhang et al. [43] found that high-voltage electrical discharges, applied as sparks in a needle-to-plate configuration, increased the extraction yields for carbohydrates, proteins, and pigments from *Nannochloropsis oculata*. The work presented here shows that also phycocyanin is extractable with competitive yields of 4 mg/g dw. This is higher than for PEF with comparable conditions, especially energy input and freeze-thawing. However, in comparison to continuous sonication with accelerated temperature development, the phycocyanin yield is much lower. Even though the temperature seems to have conductive effects to the absolute yield of raw extract, it might be detrimental to the quality of the extract. The rate of detected peptide modifications was slightly lower after spark discharge disintegration, compared to continuous sonication, indicating a more compliant extraction method. Furthermore, the purification of the raw extract resulted in a 4-time reduction of phycocyanin for the sonication extract while the purified extract after spark discharge disintegration was only reduced 1.3-times, compared to raw extract. This makes spark discharges an interesting method for industrial scaling in the field of pigment extraction from microalgae, potentially reducing the time and cost intensive purification of the raw extract.

Protein analysis was conducted to evaluate what kind of phycobiliprotein was extracted with the respective extraction method and to detect protein modifications that might be characteristic for a specific extraction method, e.g., due to temperature effects. The presence of various types of phycobiliproteins in red algae and cyanobacteria is well described in the literature [44,45,46]. Indeed, allophycocyanin (APC), C-phycocyanin (C-PC) and R-phycocyanin (R-PC) were included in all samples. The presence of phycobiliproteins after freeze-thawing detected by MS-protein analysis is in contrast to the missing characteristic blue colour of the extract, including the missing absorbance maxima. The freeze-thaw extract might contain the relevant proteins but in a degraded form, resulting in the loss of characteristic blue colour. This phenomenon is described for phycocyanin extracts after heating to temperatures exceeding 70 °C [47]. Possibly, protease activity results in the loss of blue colour after disintegration by freeze-thaw cycles. Proteolysis can lead to bleaching and some lyases might not be inactivated by the low temperature conditions [48,49]. A more detailed analysis in this field is recommended to understand the correlation of low temperatures and functional chromophore.

The fingerprint of protein distribution of the associated proteins shows no distinct deviation between the three extraction methods (spark discharge, continuous sonication, and freeze-thawing). The absence of the APC alpha chain after spark discharge disintegration was remarkable while this protein was present in both other extracts, although in low concentration. The prevalence of C-PC in the spark discharges extracts determined by protein analysis, is in good agreement with the analysis of absorbance spectra. While the maximum absorbance of APC was found at 650 nm, the C-PC absorbance peak was described within the range of 550 nm to 620 nm [50], which fits to the observed maximum absorbance after sonication and spark discharge disintegration, depicted in Figure 3. Overall, the rate of modifications in the amino acid chains of the peptides found in all samples was rather low. Notably, the extract obtained by spark discharges exhibited less modifications compared to sonication and freeze-thawing (see Figure 5A). The most commonly detected modification was the oxidation of methionine, equally distributed to all three extracts (see Figure 5B). Even though the formation of reactive species by spark discharges is well described [30,31], no enhanced oxidative effect was detected by this extraction method. Methionine is a terminal amino acid of the apoprotein and is prone to oxidation processes [51,52]. Methionine oxidation was found in all three extracts, but only the extracts obtained by sonication and spark discharges revealed a characteristic blue colour; thus, methionine oxidation seems to have no effect on the functionality of the phycocyanin. Interestingly, no modification of cysteine was found, neither for the blue coloured extracts nor for the colour-less extract obtained by freeze-thaw cycles.

The red alga *Cyanidium caldarium* comprises phycobiliproteins extractable by sonication and spark discharges. The most prevalent protein is C-phycocyanin, albeit allophycocyanin, R-phycocyanin and phycoerythrin were found likewise in both extracts. Sonication showed to be the most effective extraction method with regard to absolute yield of phycocyanin (4-times higher after purification, compared to spark discharge). However, protein analysis revealed exposure to spark discharge disintegration improved extracted protein integrity. Thus, spark discharge disintegration seems to be promising for targeted extraction of relevant high-purity phycocyanin amounts.

## 5. Conclusions

Disintegration by spark discharges, as well as disintegration by sonication facilitated the extraction of phycocyanin from the red algae *Cyanidium caldarium.* Compared to other disintegration techniques (freeze-thaw cycles and PEF), continuous sonication in particular showed to be the most effective extraction method with regard to absolute yield of phycocyanin (53 mg/g dw), although the extraction yield was lower (4 mg/g dw) in comparison to sonication, a positive effect of spark discharge disintegration towards the integrity of extracted proteins was revealed by protein analysis. Here, only minor modifications were detected, while a similar effect to such an extent was not found for sonication. Further investigations may address whether the high yield of phycobiliproteins after continuous sonication is transferrable to larger scale experiments. Adaptations of the spark discharge settings may lead to an equivalent yield with focus on a high-purity phycocyanin, saving time and material for purification.

## Figures and Tables

**Table 1:** Flow gradient for nanoLC separation. (A): water with 0.1 % *v*/*v* acetic acid, (B): acetonitrile with 0.1 % *v*/*v* acetic acid.

Eluent B	Time [min]	Flow [µL/min]
5%	0	0.300
5%	4	0.300
5%	6	0.300
35%	30	0.300
80%	33	0.300
80%	35	0.300
5%	36	0.300
5%	48	0.300

## Data Availability

Not applicable.

## Supplementary Materials

The following are available online at https://www.mdpi.com/article/10.3390/microorganisms9071452/s1, Figure S1: Pictures of raw extracts obtained by freeze-thaw cycles (a), sonication (b), and spark discharges (c).

## References

[1] Kumar D., Dhar D.W., Pabbi S., Kumar N., Walia S. (2014). Extraction and purification of C-phycocyanin from *Spirulina platensis* (CCC540). Indian J. Plant Physiol..

[2] Feketea G., Tsabouri S. (2017). Common food colorants and allergic reactions in children: Myth or reality?. Food Chem..

[3] Costa D., Mendonça M., Lopes M., Fernandes A.L., Nunes S., Müller S. (2020). Patent blue V dye anaphylaxis: A case report and literature review. Rev. Bras. Anestesiol..

[4] Ku C.S., Yang Y., Park Y., Lee J. (2013). Health benefits of blue-green algae: Prevention of cardiovascular disease and nonalcoholic fatty liver disease. J. Med. Food.

[5] Remirez D., Ledón N., González R. (2002). Role of histamine in the inhibitory effects of phycocyanin in experimental models of allergic inflammatory response. Mediat. Inflamm..

[6] Romay C., Gonzalez R., Ledon N., Remirez D., Rimbau V. (2003). C-phycocyanin: A biliprotein with antioxidant, anti-inflammatory and neuroprotective effects. Curr. Protein Pept. Sci..

[7] Jiang L., Wang Y., Yin Q., Liu G., Liu H., Huang Y., Li B. (2017). Phycocyanin: A Potential Drug for Cancer Treatment. J. Cancer.

[8] Eriksen N.T. (2008). Production of phycocyanin—A pigment with applications in biology, biotechnology, foods and medicine. Appl. Microbiol. Biotechnol..

[9] Brock T.D., Brock T.D. (1978). The Genus Cyanidium. Thermophilic Microorganisms and Life at High Temperatures.

[10] Hirooka S., Tomita R., Fujiwara T., Ohnuma M., Kuroiwa H., Kuroiwa T., Miyagishima S.Y. (2020). Efficient open cultivation of cyanidialean red algae in acidified seawater. Sci. Rep..

[11] Hu Q., Marquardt J., Iwasaki I., Miyashita H., Kurano N., Mörschel E., Miyachi S. (1999). Molecular structure, localization and function of biliproteins in the chlorophyll a/d containing oxygenic photosynthetic prokaryote Acaryochloris marina. Biochim. Biophys. Acta (BBA) Bioenerg..

[12] Ghosh T., Paliwal C., Maurya R., Mishra S., Bahadur B., Venkat Rajam M., Sahijram L., Krishnamurthy K.V. (2015). Microalgal Rainbow Colours for Nutraceutical and Pharmaceutical Applications. Plant Biology and Biotechnology: Volume I: Plant Diversity, Organization, Function and Improvement.

[13] Singh N.K., Sonani R.R., Rastogi R.P., Madamwar D. (2015). The phycobilisomes: An early requisite for efficient photosynthesis in cyanobacteria. EXCLI J..

[14] Abalde J., Betancourt L., Torres E., Cid A., Barwell C. (1998). Purification and characterization of phycocyanin from the marine cyanobacterium Synechococcus sp. IO9201. Plant Sci..

[15] Padyana A.K., Bhat V.B., Madyastha K.M., Rajashankar K.R., Ramakumar S. (2001). Crystal Structure of a Light-Harvesting Protein C-Phycocyanin from *Spirulina platensis*. Biochem. Biophys. Res. Commun..

[16] Martelli G., Folli C., Visai L., Daglia M., Ferrari D. (2014). Thermal stability improvement of blue colorant C-Phycocyanin from *Spirulina platensis* for food industry applications. Process Biochem..

[17] Mercer F.V., Bogorad L., Mullens R. (1962). Studies with Cyanidium caldarium. I. The fine structure and systematic position of the organism. J. Cell Biol..

[18] Safaei M., Maleki H., Soleimanpour H., Norouzy A., Zahiri H.S., Vali H., Noghabi K.A. (2019). Development of a novel method for the purification of C-phycocyanin pigment from a local cyanobacterial strain Limnothrix sp. NS01 and evaluation of its anticancer properties. Sci. Rep..

[19] Chittapun S., Jonjaroen V., Khumrangsee K., Charoenrat T. (2020). C-phycocyanin extraction from two freshwater cyanobacteria by freeze thaw and pulsed electric field techniques to improve extraction efficiency and purity. Algal Res..

[20] Doke J.M. (2005). An Improved and Efficient Method for the Extraction of Phycocyanin from *Spirulina* sp.. Int. J. Food Eng..

[21] Schmidt R.A., Wiebe M.G., Eriksen N.T. (2005). Heterotrophic high cell-density fed-batch cultures of the phycocyanin-producing red alga Galdieria sulphuraria. Biotechnol. Bioeng..

[22] Martinez J.M., Luengo E., Saldana G., Alvarez I., Raso J. (2017). C-phycocyanin extraction assisted by pulsed electric field from Artrosphira platensis. Food Res. Int..

[23] Martínez J.M., Delso C., Álvarez I., Raso J. (2019). Pulsed electric field permeabilization and extraction of phycoerythrin from Porphyridium cruentum. Algal Res..

[24] Luengo E., Condón-Abanto S., Álvarez I., Raso J. (2014). Effect of pulsed electric field treatments on permeabilization and extraction of pigments from Chlorella vulgaris. J. Membr. Biol..

[25] Kurokawa M., King P.M., Wu X., Joyce E.M., Mason T.J., Yamamoto K. (2016). Effect of sonication frequency on the disruption of algae. Ultrason. Sonochem..

[26] Schoenbach K.H., Peterkin F.E., Alden R.W., Beebe S.J. (1997). The effect of pulsed electric fields on biological cells: Experiments and applications. IEEE Trans. Plasma Sci..

[27] Qin S., Timoshkin I.V., Maclean M., Wilson M.P., MacGregor S.J., Given M.J., Anderson J.G., Wang T. (2014). Pulsed Electric Field Treatment of Microalgae: Inactivation Tendencies and Energy Consumption. IEEE Trans. Plasma Sci..

[28] Kotnik T., Frey W., Sack M., Haberl Meglic S., Peterka M., Miklavcic D. (2015). Electroporation-based applications in biotechnology. Trends Biotechnol..

[29] Scherer D., Krust D., Frey W., Mueller G., Nick P., Gusbeth C. (2019). Pulsed electric field (PEF)-assisted protein recovery from Chlorella vulgaris is mediated by an enzymatic process after cell death. Algal Res..

[30] Zocher K., Banaschik R., Schulze C., Schulz T., Kredl J., Miron C., Schmidt M., Mundt S., Frey W., Kolb J.F. (2016). Comparison of Extraction of Valuable Compounds from Microalgae by Atmospheric Pressure Plasmas and Pulsed Electric Fields. Plasma Med..

[31] Zocher K., Lackmann J.-W., Volzke J., Steil L., Lalk M., Weltmann K.-D., Wende K., Kolb J.F. (2019). Profiling microalgal protein extraction by microwave burst heating in comparison to spark plasma exposures. Algal Res..

[32] Zocher K., Rataj R., Steuer A., Weltmann K.D., Kolb J.F. (2020). Mechanism of microalgae disintegration by spark discharge treatment for compound extraction. J. Phys. D Appl. Phys..

[33] Locke B.R., Thagard S.M. (2012). Analysis and Review of Chemical Reactions and Transport Processes in Pulsed Electrical Discharge Plasma Formed Directly in Liquid Water. Plasma Chem. Plasma Process..

[34] Eing C., Goettel M., Straessner R., Gusbeth C., Frey W. (2013). Pulsed Electric Field Treatment of Microalgae—Benefits for Microalgae Biomass Processing. IEEE Trans. Plasma Sci..

[35] Goettel M., Eing C., Gusbeth C., Straessner R., Frey W. (2013). Pulsed electric field assisted extraction of intracellular valuables from microalgae. Algal Res..

[36] Bennett A., Bogorad L. (1973). Complementary chromatic adaptation in a filamentous blue-green alga. J. Cell Biol..

[37] UniProt Consortium UniProt: The Universal Protein Knowledgebase. https://www.uniprot.org/.

[38] Van Eykelenburg C. (1977). On the morphology and ultrastructure of the cell wall of *Spirulina platensis*. Antonie Leeuwenhoek.

[39] Sutanto H., Suzery M. (2016). Phyocyanin extraction from microalgae *Spirulina platensis* assisted by ultrasound irradiation: Effect of time and temperature. Songklanakarin J. Sci. Technol..

[40] Li Y., Zhang Z., Paciulli M., Abbaspourrad A. (2020). Extraction of phycocyanin-A natural blue colorant from dried spirulina biomass: Influence of processing parameters and extraction techniques. J. Food Sci..

[41] Fratelli C., Burck M., de Amarante M.C.A., Braga A.R.C. (2020). Antioxidant potential of nature’s “something blue”: Something new in the marriage of biological activity and extraction methods applied to C-phycocyanin. Trends Food Sci. Technol..

[42] Akaberi S., Krust D., MŘller G., Frey W., Gusbeth C. (2020). Impact of incubation conditions on protein and C-Phycocyanin recovery from Arthrospira platensis post-pulsed electric field treatment. Bioresour. Technol..

[43] Zhang R., Marchal L., Lebovka N., Vorobiev E., Grimi N. (2020). Two-step procedure for selective recovery of bio-molecules from microalga Nannochloropsis oculata assisted by high voltage electrical discharges. Bioresour. Technol..

[44] Apt K.E., Collier J.L., Grossman A.R. (1995). Evolution of the Phycobiliproteins. J. Molecular Biol..

[45] Kremer B.P., Feige G.B. (1979). Accumulation of Photoassimilatory Products by Phycobiliprotein- Containing Algae with Special Reference to Cyanidium caldarium. Z. Naturforschung C.

[46] Troxler R.F., Ehrhardt M.M., Brown-Mason A.S., Offner G.D. (1981). Primary structure of phycocyanin from the unicellular rhodophyte Cyanidium caldarium. II. Complete amino acid sequence of the beta subunit. J. Biol. Chem..

[47] Böcker L., Ortmann S., Surber J., Leeb E., Reineke K., Mathys A. (2019). Biphasic short time heat degradation of the blue microalgae protein phycocyanin from Arthrospira platensis. Innov. Food Sci. Emerg. Technol..

[48] Ocarra P. (1965). Purification and N-terminal analysis of algal biliproteins. Biochem. J..

[49] Scheer H., Zhao K.H. (2008). Biliprotein maturation: The chromophore attachment. Mol. Microbiol..

[50] Stadnichuk I.N. (1995). Phycobiliproteins: Determination of chromophore composition and content. Phytochem. Anal..

[51] Romay C., Gonzalez R., Pizarro M., Lissi E. (2000). Kinetics of c-phycocyanin reaction with hypochlorite. J. Protein Chem..

[52] Stec B., Troxler R.F., Teeter M.M. (1999). Crystal Structure of C-Phycocyanin from Cyanidium caldarium Provides a New Perspective on Phycobilisome Assembly. Biophys. J..

