# Supplementary material for: Assessment of Phycocyanin Extraction from Cyanidium caldarium by Spark Discharges, Compared to Freeze-Thaw Cycles, Sonication, and Pulsed Electric Fields
Source: Microorganisms. 2021 Jul 6;9(7):1452. doi: 10.3390/microorganisms9071452 (PMC8303284; doi:10.3390/microorganisms9071452)
Supplement: Supplementary file 1 [file microorganisms-09-01452-s001.zip › microorganisms-1288900-supplementary.pdf]

Supplementary Materials

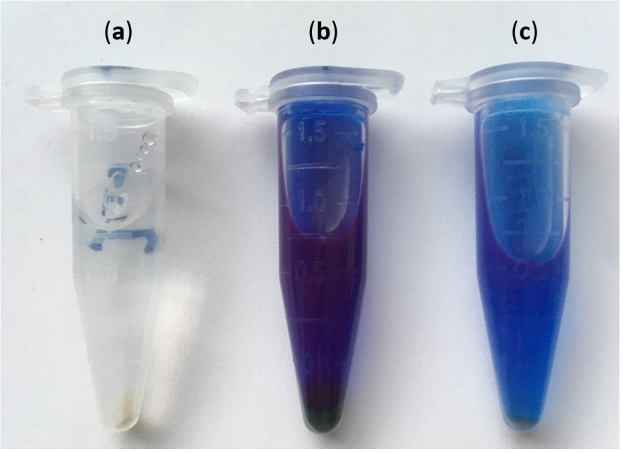

Figure S1. Pictures of raw extracts obtained by freeze-thaw cycles (**a**), sonication (**b**), and spark discharges (**c**).
